# CONTENDING WITH UNCERTAINTY: IMPLEMENTING THE CMS ACUTE HOSPITAL CARE AT HOME WAIVER PROGRAM IN THE UNITED STATES

**DOI:** 10.1093/geroni/igac059.994

**Published:** 2022-12-20

**Authors:** Ksenia Gorbenko, Emily Franzosa, Abigail Baim-Lance, Gabrielle Schiller, Heather Wurtz, Sybil Masse, David Levine, Albert Siu

**Affiliations:** Icahn School of Medicine at Mount Sinai, New York, New York, United States; Icahn School of Medicine at Mount Sinai, New York, New York, United States; Icahn School of Medicine at Mount Sinai, New York, New York, United States; CUNY School of Public Health, Astoria, New York, United States; Icahn School of Medicine at Mount Sinai, New York, New York, United States; Icahn School of Medicine at Mount Sinai, New York, New York, United States; Mass General Brigham, Boston, Massachusetts, United States; Icahn School of Medicine at Mount Sinai, New York, New York, United States

## Abstract

As Congress considers renewing the Acute Hospital Care At Home (AHCaH) waiver, which provides a full hospital payment for Hospital at Home (HaH) care, evaluating uncertainty around the future of HaH payment is critical. Our qualitative study explored HaH leaders’ experiences with implementing HaH (N=18, clinical/medical directors, operational and program managers) from 14 new and pre-existing programs across the U.S. We conducted semi-structured interviews with HaH programs diverse by size, urbanicity, and geography. We analyzed transcripts using a thematic approach. Participants across settings and regions wanted greater clarity about the waiver’s future. Lack of clarity affected staffing (nurses reluctant to take temporary jobs) and investment in establishing programs (building EMR components, changing workflows, creating inpatient processes in an outpatient setting). Programs adapted to uncertainty in multiple ways: 1) operating parallel waiver and non-waiver programs; 2) seeking to determine/ calculate the HaH value for their institution; 3) determining which patients would benefit most from HaH; and 4) seeking additional health system financing options beyond the CMS reimbursement (new programs) or relying on existing contracts with payers (existing programs). Implementing HaH is a complex and resource intensive process. Greater clarity from CMS regarding the waiver’s future state will encourage programs to invest the resources that they need to establish their programs long-term. Waiver extension/ permanence would also enable programs to develop and test measures of value, making rigorous evaluations possible to optimize different HaH components.

